# The Influenza

**Published:** 1891-06-06

**Authors:** May Yates

**Affiliations:** Org. Sec., London Vegetarian Society, Memorial Hall, Farringdon Street, E.C.


### EDITOR'S LETTER-BOX.

### THE INFLUENZA.

Sir,—As the above epidemic is so very prevalent will you kindly allow me to direct the attention of your readers to the value of a large consumption of fresh fruit as a means of enabling the system to resist infection and diminishing the danger of the disease when it has been incurred. Oranges and all fruits in their natural uncooked condition are especially valuable, as there is good reason to believe that natural foods contain an electric or potential energy, which, as the chemical ingredients of food supply the chemical constituents of the body, may increase human energy. The whole subject of electric, magnetic, and other forms of energy and force is so imperfectly understood that it is impossible to dogmatise on this subject; but, apart from any theory on the subject, I can personally testify to the practical benefits derived from the use of a large amount of fresh fruit. Last year I had a bad attack of influenza, but felt none of the distressing weakness of which so many complained, and attribute this to the fact that I took no meat, but plenty of fruit combined with whole meal, lentils, nuts, milk, and eggs. At the beginning of the present epidemic I had every sign of having caught the infection, but the consumption of a large amount of oranges and grapes at once stopped it. The prevalence of the disease amongst the wealthy classes appears to indicate that the excrementitious elements contained in the meat, which forms so large a portion of their diet, affords a highly favourable nidus for the development of the influenza microbe. The experience of vegetarians indicates that a properly-selected vegetarian diet increases the resisting power of the constitution, and by keeping the blood in a pure condition, makes any attacks of the disease of a mild and simple nature. I therefore venture at the present time to direct the special attention of your readers to this subject, and I shall be pleased (on receipt of a directed wrapper) to send report and explanatory leaflets.—Yours truly, May Yates,

Org. Sec., London Vegetarian Society,

Memorial Hall, Farringdon Street, E.C.

[This letter is criticised on p. 116.—Ed.]

